# Efficacy and Safety of Andexanet Alfa Versus Four Factor Prothrombin Complex Concentrate for Emergent Reversal of Factor Xa Inhibitor Associated Intracranial Hemorrhage: A Systematic Review and Meta-Analysis

**DOI:** 10.1007/s12028-024-02130-y

**Published:** 2024-10-08

**Authors:** Khalid Sarhan, Rashad G. Mohamed, Reem Reda Elmahdi, Youstina Mohsen, Asmaa Elsayed, Dania Mosaad Zayed, Menna A. Elkholi, Nagat Gabr, Enjy M. El-Bialy, Ibrahim Serag

**Affiliations:** 1 Faculty of Medicine, Mansoura University, Mansoura, Egypt; 2 Mansoura Manchester Program for Medical Education, Faculty of Medicine, Mansoura University, Mansoura, Egypt

**Keywords:** Andexanet alfa, Prothrombin complex concentrates, Anticoagulants, Intracerebral hemorrhage, Reversal agents

## Abstract

Factor Xa inhibitors (FXaI) are increasingly used for anticoagulation therapy, yet their association with intracranial hemorrhage poses a significant challenge. Although andexanet alfa (AA) and four-factor prothrombin complex concentrate (4F-PCC) have shown promise in reversing FXaI effects, their comparative efficacy and safety remain uncertain. Following the Preferred Reporting Items for Systematic Reviews and Meta-Analyses guidelines, we conducted a literature search on electronic databases to obtain the relevant studies until May 16, 2024. Our primary outcomes were successful anticoagulation reversal, overall mortality (including 30-day and in-hospital mortality), and thromboembolic events. Secondary outcomes were length of hospital and intensive care unit stay and hematoma volume expansion. Data were pooled using a random-effects model. We included 16 eligible studies with a total of 2,977 patients. A statistically significant improvement in hemostatic efficacy rates was in favor of the AA group (risk ratio [RR] 1.10, 95% confidence interval [CI] 1.01–1.20, *P* = 0.02). Lower overall mortality rates were found in the AA group (RR 0.67, 95% CI 0.51–0.88, *P* = 0.004). However, no difference was found in 30-day mortality rates (RR 0.82, 95% CI 0.58–1.16, *P* = 0.26). In terms of thromboembolic events, more events were found in the AA group (RR 1.47, 95% CI 1.01–2.15, *P* = 0.046). AA was associated with a longer duration of hospital stay compared to 4F-PCC (mean difference [MD] 0.64, 95% CI 0.07–1.22, *P* = 0.03). Neither a significant difference in length of intensive care unit stay (MD 0.25, 95% CI − 0.36 to 0.86, *P* = 0.41) nor a significant difference in hematoma volume expansion was reported (MD − 0.89, 95% CI − 3.11 to 1.34, *P* = 0.435). Our results suggest that AA is superior to 4F-PCC in enhancing the hemostatic efficacy and reducing the overall and in-hospital mortality rates. More thromboembolic events are thought to be associated with the use of AA. However, more studies are required to validate whether the better results of AA in improving hemostatic efficacy are enough to make up for their higher cost and their possible risk of thromboembolic events.

**Supplementary Information:**

The online version contains supplementary material available at 10.1007/s12028-024-02130-y.

## Introduction

Factor Xa inhibitors (FXaI), such as apixaban, rivaroxaban, edoxaban, and betrixaban, are types of non–vitamin K oral anticoagulants or direct oral anticoagulants (DOACs) [1, 2]. The US Food and Drug Administration (FDA) has approved these agents for the prevention of many thromboembolic diseases, such as stroke prevention in atrial fibrillation, prophylaxis after major orthopedic surgeries, and treatment of venous thromboembolism, whereas some of them have more specific indications [1]. DOACs are increasingly used nowadays in clinical practice because they have proven in several studies to provide comparable efficacy and superior safety in lowering the risk of intracranial hemorrhage (ICH) when compared to traditional agents, such as warfarin [3–6]. However, the management of a life-threatening ICH in patients receiving oral FXaI is a critical challenge in emergency medicine because rapid and effective reversal is required to help stop or reduce the bleeding.

Before the approval of andexanet alfa (AA) in May 2018 by the FDA as a specific reversal agent for FXaI [7], the reliance on nonspecific agents, such as fresh frozen plasma, activated prothrombin complex concentrate (aPCC), and four-factor prothrombin complex concentrate (4F-PCC), was a considerable viable option, although their efficacy was not well established [8, 9]. AA has received accelerated approval after the results of two phase III trials from the ANNEXA program, which demonstrated a significant decrease in anti-factor Xa activity from baseline [10, 11]. However, factors such as medication cost and safety concerns were considered limitations for the use of AA. It is estimated that the national average hospital reimbursement per patient will be exceeded by more than $7,500/hospitalization for up to 75% of patients who receive AA for oral FXaI reversal [12]. Clinical outcome reviews associated with the therapy in clinical practice are necessary to determine whether the higher cost of AA therapy compared to 4F-PCC is justified by its greater effectiveness. These reviews should also investigate whether the choice of drug should be tailored based on risk factors for poor outcomes and hematoma expansion in patients experiencing major bleeding [13]. There have been differing reports regarding the safety profiles of AA and 4F-PCC in reversing FXaI, particularly concerning mortality rates and thromboembolic events [14–17]. However, these comparisons are restricted to data from cohort studies. ANNEXA-1 is the first randomized trial to assess the safety and efficacy of AA compared with usual care, mostly prothrombin complex concentrate in patients with intracerebral hemorrhage [18]. To the best of our knowledge, only one previous meta-analysis compared AA with 4F-PCC, in which all the included studies were limited to cohort studies and no significant difference was found between both groups. To overcome these limitations, we conducted this systematic review and meta-analysis, including the results of the ANNEXA-1 trial, to better guide clinical decision making and provide insights into the comparative efficacy and safety of AA and 4F-PCC in the emergent reversal of FXaI-associated ICH.

## Methods

### Protocol

This systematic review and meta-analysis were performed in accordance with the established guidelines outlined by Preferred Reporting Items for Systematic Reviews and Meta-Analyses (PRISMA) [19]. The protocol was registered in PROSPERO (CRD42024525996). All authors worked independently on the study retrieval, eligibility evaluation, data extraction, and quality assessment.

### Search Strategy

We thoroughly searched four databases: PubMed, Scopus, Web of Science, and the Cochrane Library, until May 16, 2024, for eligible studies. No limitations were placed on publication status or geographical areas. The search strategy was conducted using medical subject headings and their equivalents. The search terms used were “Prothrombin complex concentrates,” “Andexanet Alfa,” “Factor Xa Inhibitor,” and “intracranial hemorrhage.” The vocabulary and syntax were modified in accordance with the needs of each database, and the Boolean operators “AND” and “OR” were employed to join the terms. The detailed search strategy for the four databases is shown in (eAppendix 1).

### Eligibility Criteria

Only clinical studies that met the following requirements were included in this study: (1) any randomized or nonrandomized control trials; (2) cohort studies (prospective or retrospective) and case control studies with adult patients presenting with life-threatening traumatic or spontaneous intraparenchymal, subarachnoid, and subdural hemorrhages and other intracranial bleeds; (3) FXaI used at baseline; (4) and AA or 4F-PCC received for reversal of ICH. Expansion records were excluded if they were (1) non-English; (2) other study designs, such as case reports, case series, nonmedical articles, reviews, conference abstracts, animal or in vitro studies, and preclinical studies; (3) studies involving other anticoagulant reversal drugs (e.g., aPCC) or the use of both prothrombin complex concentrate and AA together for anticoagulant reversal; and (4) studies including any other source of injury or hemorrhage aside from intracranial bleeding.

### Outcome Definition

Our primary efficacy and safety outcomes were successful reversal of anticoagulation, all-cause mortality (including in-hospital and 30-day mortality subgroups), and in-hospital thromboembolic events following administration of AA or 4F-PCC. Our secondary outcomes were duration of hospital stay, duration of intensive care unit (ICU) stay, hematoma volume expansion, and good clinical outcome (defined as modified Rankin scale [mRS] score of 0 to 3 or Glasgow Outcome Scale [GOS] score of more than 3).

### Study Selection

**Fig. 1:**
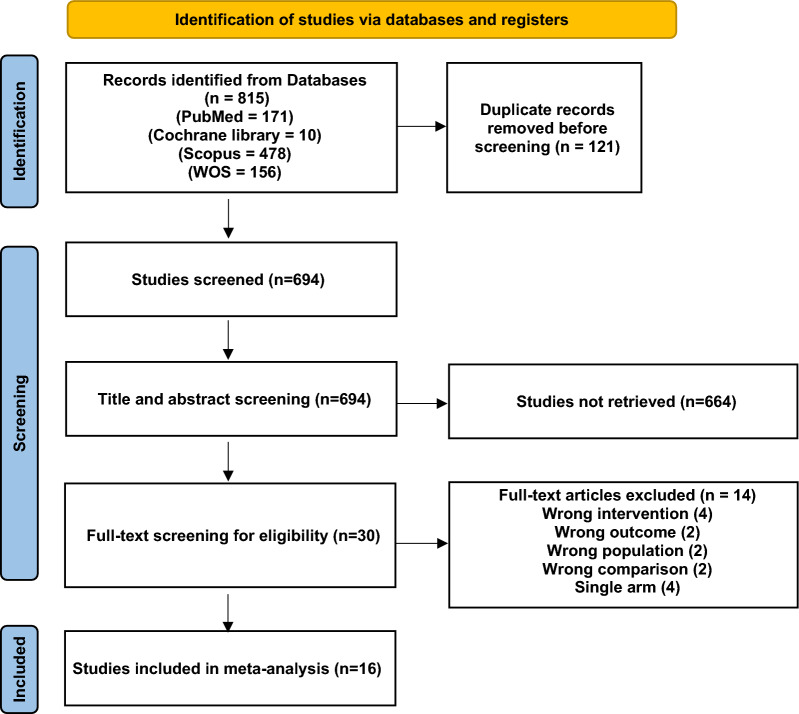
Prisma flow diagram of the included studies

After removing duplicates using Endnote X9, two researchers independently reviewed each study’s title and abstract to exclude any irrelevant articles based on our eligibility criteria. Then the full text of studies that met our predetermined inclusion criteria were screened. Any disagreements were settled by discussion and agreement with a third reviewer. A PRISMA flow diagram of the included studies is shown in Fig. 1.

### Quality Assessment and Publication *Bias*

Two independent researchers used the Newcastle–Ottawa Quality Assessment Scale to evaluate the quality of the included cohort studies, in which three broad parameters were used to evaluate each study: selection and representativeness of the study groups, comparability of the groups, and the ascertainment of either the exposure or outcome of interest [20]. Study quality was categorized into one of three groups according to the following score ranges: 0 to 3, 4 to 6, and 7 to 9. These ranges corresponded to low, medium, and high study quality, respectively. The ROB-2 tool was used to assess the quality of the only randomized controlled trial, the ANNEXA-1 trial [18]. Using a funnel plot with ten or more included studies, publication bias was virtually investigated. The Egger test [21] and Begg-Mazumdar test [22] were used to quantify any additional bias that was observed.

### Statistical Analysis

Risk ratio (RR) and 95% confidence interval (CI) were computed for dichotomous variables (e.g., successful anticoagulation reversal, in-hospital thrombotic events during reversal therapy, and mortality). For continuous variables (e.g., length of hospital and ICU stay and hematoma volume expansion), the mean difference (MD) and 95% CI were computed to pool effect estimates. The method by Wan et al. [23] was used to convert any outcome reported in the form of median and interquartile range (IQR) to mean and standard deviation. We used a random-effects model because of the expected heterogeneity between studies. Higgins *I*^2^ test was used to measure heterogeneity, in which a range of 0–40% was deemed insignificant, a range of 30–60% was considered moderate, a range of 50–90% represented substantial, and a range of 75–100% represented considerable heterogeneity [24]. Sensitivity analyses were employed to evaluate the result’s robustness and to assess the contribution of each study by excluding studies each at a time. We conducted our statistical analysis using Review Manager V.5.3 [25] and Open Meta-Analyst software [26].

## Results

### Study Selection

Our search retrieved 815 studies. Following title and abstract screening, 29 studies were eligible for full-text screening. A total of 16 studies with 2,977 patients met the eligibility criteria and were included in this meta-analysis. Of the 16 studies comparing AA and 4F-PCC in patients with ICH while receiving FXaI, 1,560 patients used 4F-PCC, and 1,417 patients used AA as a reversal agent. A summary of the included studies is presented in (eAppendix 2).

### Baseline Characteristics

Overall, the mean (range) age was 77 years (65–84 years), and 58% were male. The mean (range) systolic blood pressure was 144.6 mm Hg (138–161 mm Hg) in the AA group and 144.3 mm Hg (131–159 mm Hg) in the 4F-PCC group. The median (IQR) door-to-reversal time was 1.8 h (1.1–2.4 h) in the AA group and 1.2 h (0.7–2.3 h) in the 4F-PCC group. The most used FXaI were apixaban and rivaroxaban, and they were mostly indicated for atrial fibrillation and venous thromboembolism (eAppendix 3). The baseline characteristics of the included studies are presented in Table 1.The type and cause of ICH are presented in Table 2.

**Table 1:** Baseline characteristics of the included studies

Study ID	Sample size		Age, mean (SD)		Female sex, *n* (%)		GCS at baseline, mean (SD)		BMI, mean (SD)		Systolic blood pressure, mean (SD)		Door-to-reversal time (hours), median (IQR)	
	AA	4F-PCC	AA	4F-PCC	AA	4F-PCC	AA	4F-PCC	AA	4F-PCC	AA	4F-PCC	AA	4F-PCC
Ammar et al. [14]	28	16	78.3 (13.3)	79.3 (8.1)	11 (39%)	5 (31%)	13 (3.12)	12 (6.50)	31.3 (17.2)	29 (5.7)	NA	NA	NA	NA
ANNEXA-I [18]	224	228	78.9 (8.5)	78.9 (8.5)	94 (42%)	113 (49.6%)	14.33 (1.49)	14.33 (1.49)	26.9 (5.3)	26.3 (4.6)	161.2 (27)	159.8 (27.7)	2.1 (1.5–2.9)	2.3 (1.7–3.1)
Barra et al. [29]	18	11	80.8 (14.9)	70.9 (3.9)	8 (44%)	2 (18%)	14.67(0.80)	9.67 (6)	25.9 (6.8)	31 (4.2)	NA	NA	3.6 (2.7–5.0)	2.6 (2.0–5.0)
Coleman et al. [30]	67	170	NA	NA	NA	NA	NA	NA	NA	NA	NA	NA	NA	NA
Costa et al. [27]	107	95	79 (8)	79 (11)	54 (50%)	48 (50%)	14 (1)	14 (2)	28 (7)	28 (6)	NA	NA	NA	NA
Dobesh et al. [28]	666	662	NA	NA	NA	NA	NA	NA	NA	NA	139.1 (61.2)	131.2 (61.8)	2.5 (1.2–6.4)	2.3 (1.2–5.7)
Irizarry-Gatell et al. 2024 [31]	23	66	77 (12.6)	76.8 (9.8)	5 (22%)	34 (52%)	13 (2.4)	12 (3.8)	86.4 (22.3)	79.1 (21.2)	NA	NA	NA	NA
Koo et al. [32]	58	41	NA	NA	NA	NA	NA	NA	NA	NA	NA	NA	0.76 (0.55–1.08)	0.72 (0.5–1.13)
Lipski et al. [33]	23	47	79.3 (9.5)	80.6 (9.9)	13 (28%)	17 (36%)	10.33(7.11)	11.3 (6.9)	29.7 (6.5)	27.7 (6.5)	NA	NA	NA	NA
Milioglou et al. [34]	23	22	76 (8)	77 (7)	10 (43%)	8 (36%)	NA	NA	83.1 (18)	87.5 (15)	NA	NA	3.3 (1.8–2.7)	2.3 (1.8–2.7)
Oh et al. [35]	9	15	79.3 (12.2)	84 (10.6)	5 (56%)	6 (40%)	14.67(0.87)	14.6 (0.8)	30.2 (11.1)	27.0 (5.15)	159.3 (15.7)	138.3 (26.7)	1.32 (1.09–2.26)	2.31 (1.21–3.29)
Parsels et al. [16]	26	26	80.7 (12.1)	78.2 (13.4)	16 (61%)	11 (42%)	13.83(2.35)	14.4 (0.9)	78.1 (32.1)	80.5 (26)	138 (28.2)	148 (30.7)	NA	NA
Pham et al. [15]	47	62	77.6 (12.2)	79.3 (11.4)	16 (34%)	30 (48%)	13.0 (3.82)	14.0 (1.5)	NA	NA	NA	NA	1.16 (0.9–1.45)	0.71 (0.51–1.02)
Stevens et al. [36]	7	10	NA	NA	NA	NA	8.67(5.51)	13 (3.4)	NA	NA	NA	NA	NA	NA
Troyer et al. [37]	31	15	77.8 (10.5)	70.3 (6.54)	13 (42%)	9 (40%)	12.5 (3.85)	10.6 (5.2)	NA	NA	NA	NA	NA	NA
Vestal et al. [17]	37	289	73.3 (12)	74.5 (3.4)	6 (29%)	21(60%)	14.08(1.79)	11.6 (6.2)	81.9 (22)	82.6 (24.6)	NA	NA	2.67 (1.75–4.13)	1.73 (1.21–3.55)

*4F-PCC* four-factor prothrombin complex concentrate, *AA* andexanet alfa, *BMI* body mass index, *GCS* Glasgow Coma Scale, *IQR* interquartile range, *NA* not available

**Table 2:** Type and cause of ICH in each of the included studies

Study ID	Type of ICH								Cause of bleeding			
	AA				4F-PCC				AA		4F-PCC	
	ICH N/T	SAH N/T	SDH N/T	Others N/T	ICH N/T	SAH N/T	SDH N/T	Others N/T	Spontaneous onset N/T	Traumatic onset N/T	Spontaneous onset N/T	Traumatic onset N/T
Ammar et al. [14]	0/28	2/28	1/28	25/28	0/16	0/16	0/16	13/16	20/28	8/28	8/16	8/16
ANNEXA-I [18]	198/224	9/224	13/224	4/224	214/224	8/228	4/228	2/228	202/224	22/224	195/228	33/228
Barra et al. [29]	0/8	1/18	5/18	12/18	0/8	0/11	3/11	8/11	6/18	12/18	6/11	5/11
Coleman et al. [30]	NA	NA	NA	NA	NA	NA	NA	NA	NA	NA	NA	NA
Costa et al. [27]	64/107	37/107	41/107	0/107	58/95	33/95	36/95	46/95	50/107	57/107	34/95	61/95
Dobesh et al. [28]	110/666	151/666	217/666	228/666	129/662	141/662	214/662	239/662	339/666	327/666	296/666	366/662
Irizarry-Gatell et al. [31]	NA	NA	NA	NA	NA	NA	NA	NA	NA	NA	NA	NA
Koo et al. [32]	NA	NA	NA	NA	NA	NA	NA	NA	NA	NA	NA	NA
Lipski et al. [33]	NA	NA	NA	NA	NA	NA	NA	NA	8/23	15/23	15/47	32/47
Milioglou et al. [34]	19/23	0/23	4/23	0/23	17/22	1/22	4/22	0/22	NA	NA	NA	NA
Oh et al. [35]	0/9	4/9	4/9	6/9	0/15	0/15	7/15	9/15	NA	NA	NA	NA
Parsels et al. [16]	10/26	9/26	5/26	2/26	9/26	11/26	5/26	1/26	10/26	16/26	8/26	18/26
Pham et al. [15]	0/47	12/47	14/47	46/47	0/62	13/62	23/62	58/62	NA	NA	NA	NA
Stevens et al. [36]	NA	NA	NA	NA	NA	NA	NA	NA	NA	NA	NA	NA
Troyer et al. [37]	NA	NA	NA	NA	NA	NA	NA	NA	NA	NA	NA	NA
Vestal et al. [17]	17/21	2/21	2/21	25/28	18/35	6/35	1/35	10/35	23/28	5/28	24/35	11/35

*4F-PCC* four-factor prothrombin complex concentrate, *AA* andexanet alfa, *ICH* intracerebral hemorrhage, *N* number, *NA* not available, *SAH* subarachnoid hemorrhage, *SDH* subdural hemorrhage, *T* total

### Risk-of-*bias* Assessment and Publication *Bias*

The ANNEXA-1 trial [18] was judged as low risk using the ROB-2 tool. However, all eligible cohort studies were deemed as high risk owing to unadjusted analysis and differences of prognostic factors and comorbidities among study groups, except the studies by Costa et al. [27] and Dobesh et al. [28], which were judged as low risk (eAppendix 2). To ensure the robustness of our results, we conducted a sensitivity analysis, which revealed that all studies drive our results (eAppendix 5). Using funnel plots, no significant publication bias was observed in the primary safety and efficacy outcomes (anticoagulation reversal, mortality, and thromboembolic events) as well as secondary outcomes. (eAppendix 6).

### Results of the Primary Outcomes

#### Anticoagulation Reversal

**Fig. 2:**
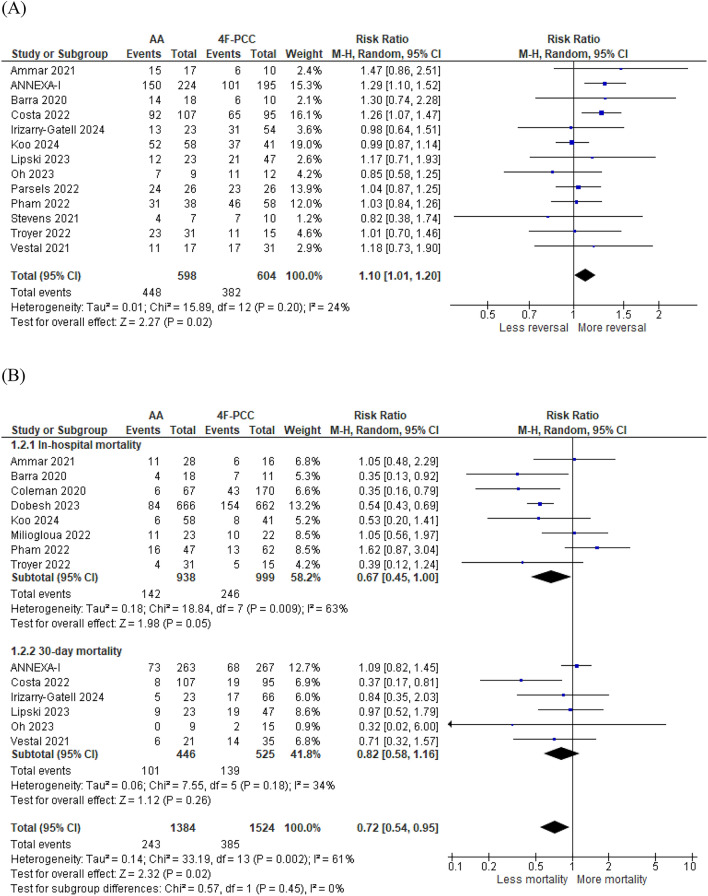

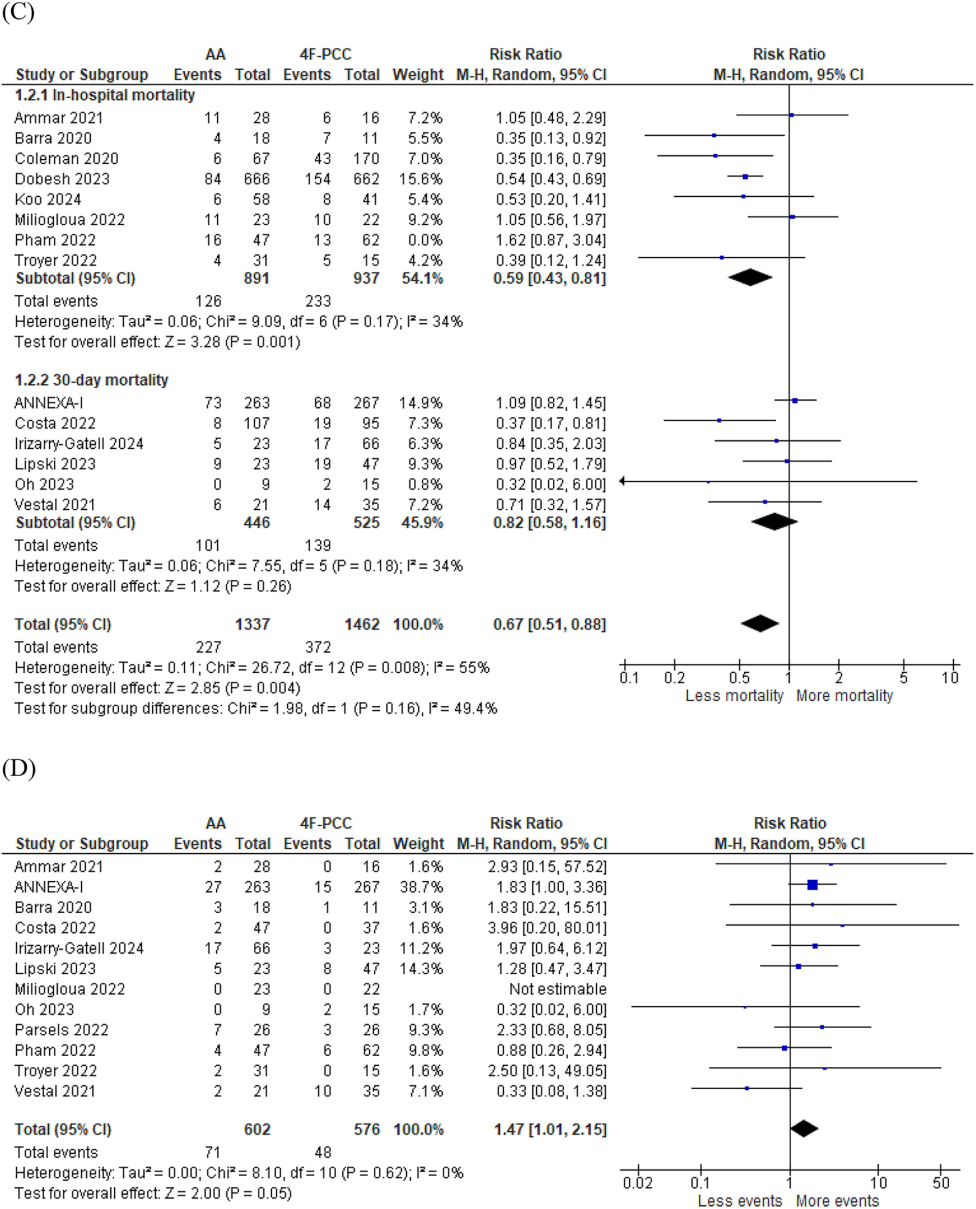
Forest plots of primary outcomes comparing andexanet alfa (AA) vs. four-factor prothrombin complex concentrate (4F-PCC). **a** Forest plot of successful anticoagulation reversal. **b** Forest plot of overall mortality, in-hospital mortality and 30-day mortality. **c** Forest plot of mortality after excluding study by Pham et al. (2022) [15] to resolve heterogeneity. **d** Forest plot of thromboembolic events

Thirteen studies including 1,202 patients compared the efficacy of 4F-PCC and AA for successful anticoagulant reversal. Hemostatic efficacy occurred in 448 of 598 patients (80.2%) in the AA group and in 382 of 604 patients (63.2%) in the 4F-PCC group. A statistically significant improvement in hemostatic efficacy was in favor of the AA group (RR 1.10, 95% CI 1.01–1.20, *P* = 0.02). No heterogeneity was found between the effects sizes of the included studies (*P* = 0.2, *I*^2^ = 24%) (Fig. 2a).

#### Mortality and Thromboembolic Events

Fourteen studies including 2,908 patients compared the safety of AA and 4F-PCC in terms of mortality rate. Death occurred in 243 of 1,384 and 385 of 1,524 patients treated with AA and 4F-PCC, respectively. There was a significant reduction in the overall mortality rate for AA versus 4F-PCC (RR 0.72, 95% CI 0.54–0.95, *P* = 0.02). However, this pooled effect was subject to uncertainty in estimation because of the presence of high heterogeneity between the studies (*P* = 0.002, *I*^2^ = 61%) (Fig. 2b). In attempt to resolve this heterogeneity and to ensure the robustness of our results, we conducted a subgroup analysis assessing 30-day mortality and in-hospital mortality individually. In the in-hospital mortality subgroup, lower mortality rates were found in the AA group (RR 0.67, 95% CI 0.45–0.99, *P* = 0.047). Significant heterogeneity was found in this subgroup (*P* = 0.009, *I*^2^ = 63%). To resolve this heterogeneity, we conducted a sensitivity analysis in multiple scenarios, excluding one study in each scenario. Heterogeneity was best resolved by excluding the study by Pham et al. [15] (*P* = 0.17, *I*^2^ = 34%). After removing this study from the meta-analysis model, the results of the in-hospital mortality analysis were still in favor of the AA group (RR 0.59, 95% CI 0.43–0.81, *P* = 0.001), and the results of the overall mortality analysis were still in favor of the AA group (RR 0.67, 95% CI 0.51–0.88, *P* = 0.004) (Fig. 2c). In the 30-day mortality subgroup, no significant difference was found between AA and 4F-PCC (RR 0.82, 95% CI 0.58–1.16, *P* = 0.26). No heterogeneity was found between the studies in this subgroup (*P* = 0.18, *I*^2^ = 34%). Twelve studies including 1,178 patients assessed the incidence of thromboembolic events, and more events were found in the AA group; however, the results were marginally significant (RR 1.47, 95% CI 1.01–2.15, *P* = 0.046). No heterogeneity was found between the studies (*P* = 0.62, *I*^2^ = 0%) (Fig. 2d).

### Results of the Secondary Outcomes

#### Length of Hospital and ICU Stay

**Fig. 3:**
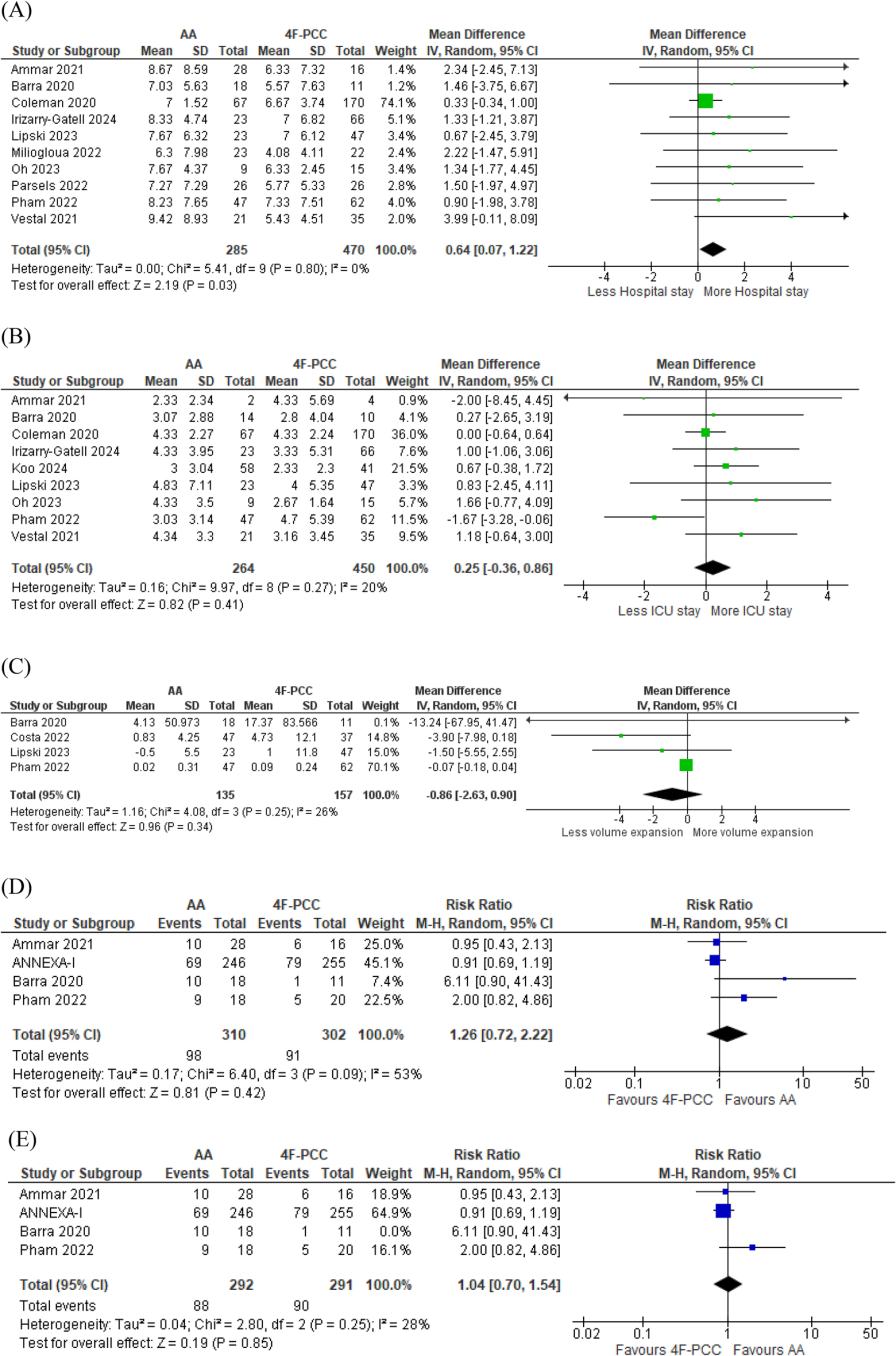
Forest plots of secondary outcomes comparing andexanet alfa (AA) vs. four-factor prothrombin complex concentrate (4F-PCC). **a** Forest plot of length of hospital stay. **b** Forest plot length of intensive care unit (ICU) admission. **c** Forest plot hematoma volume expansion. **d** Forest plot of good clinical outcome (modified Rankin scale [mRS] score ≤ 3 or Glasgow Outcome Scale [GOS] score > 3). **e** Forest plot of good clinical outcome (mRS score ≤ 3 or GOS score > 3) after exclusion of study by Barra et al. (2020) [29] to resolve heterogeneity

Ten studies including 755 patients and nine studies including 714 patients assessed the length of hospital stay and ICU stay, respectively. Length of hospital stay was more in the AA group (MD 0.64, 95% CI 0.07–1.22, *P* = 0.03). No heterogeneity was found between the effect sizes (*P* = 0.80, *I*^2^ = 0%) (Fig. 3a). Length of ICU stay was more in the AA group; however, the MD was not statistically significant between both groups (MD 0.25, 95% CI − 0.36 to 0.86, *P* = 0.41) (Fig. 3b).

#### Hematoma Volume Expansion

Four studies including 292 patients assessed hematoma volume expansion in the two groups. Less volume expansion was observed in the AA group; however, the results were not statistically significant (MD − 0.89, 95% CI − 3.11 to 1.34, *P* = 0.435) (Fig. 3c).

#### Good Clinical Outcome (mRS score ≤ 3 or GOS score > 3)

Four studies including 583 patients measured good clinical outcome (at discharge in three studies and at 30 days in ANNEXA-1 trial), which was defined as an mRS score ≤ 3 in three studies and a GOS score > 3 in one study. Good clinical outcome did not differ significantly between AA and 4F-PCC groups (RR 1.26, 95% CI 0.72–2.22, *P* = 0.42). Moderate heterogeneity was found between studies (*P* = 0.09, *I*^2^ = 53%), which was best resolved by the exclusion of the study by Barra et al. [29], the only study using GOS score > 3 as a definition of good clinical outcome. By excluding this study from the analysis model, the heterogeneity was resolved (*P* = 0.25, *I*^2^ = 28%); however, the overall results were still not favoring either of the groups (RR 1.04, 95% CI 0.7–1.54, *P* = 0.85).

## Discussion

In this systematic review and meta-analysis, we evaluated the efficacy and safety of AA versus 4F-PCC as a reversal agent for ICH in patients on FXaI. Our analysis of 16 studies with 2,977 patients revealed that the AA group had higher efficacy in successful anticoagulation reversal than the 4F-PCC group (*P* = 0.02). Also, AA has been associated with lower overall and in-hospital mortality rates. However, no difference was found in 30-day mortality rates between the two groups. In terms of thromboembolic events, more events were found in the AA group, although our results were marginally significant (*P* = 0.046). Although secondary outcomes, such as the length of ICU stay and hematoma volume expansion, did not significantly differ between the two groups, patients treated with AA tended to have a slightly longer hospital stay.

Comparing our study with the study conducted by Chaudhary et al. [38], we compared the efficacy and safety of 4F-PCC and AA in reversing anticoagulation in patients with ICH. Our findings showed a higher success rate of AA in reversing anticoagulation (RR 1.10, 95% CI 1.01–1.20, *P* = 0.02) than that reported by Chaudhary et al. (RR 0.95, 95% CI 0.85–1.06), which did not reach a statistically significant difference between both groups. Also, our analysis showed a statistically significant difference for AA in lowering overall mortality rates (RR 0.67, 95% CI 0.51–0.88, *P* = 0.004), whereas the results from the study by Chaudhary et al. failed to note this difference (RR 1.40, 95% CI 0.68–2.86). Finally, regarding the safety outcome, thromboembolic events were marginally significant between the two agents in our study (RR 1.47, 95% CI 1.01–2.15, *P* = 0.046), whereas in the study by Chaudhary et al., no significant difference was found [38].

The age distribution across the included studies shows that most patients were elderly, with a mean age of 77 years. Advanced age is a known risk factor for both ICH and adverse outcomes following anticoagulation reversal. The comorbidities commonly observed in this population, such as hypertension, may have exacerbated the severity of hemorrhage and influenced the response to reversal agents [14, 18]. Additionally, the time from anticoagulation reversal to intervention (door-to-reversal time) was a significant variable. The shorter reversal time observed in the 4F-PCC group compared to the AA group may have contributed to differences in outcomes, particularly in the context of acute ICH management [15, 32]. In our analysis, hypertension was one of the most common comorbidities among patients with ICH, as also observed in the studies by Dobesh et al. [28] and Oh et al. [35]. Elevated systolic blood pressure at baseline, particularly in patients treated with AA, was associated with higher hemostatic challenges, given that hypertension is a critical factor in both the risk and management of hemorrhagic events. In the study by Dobesh et al. [28], patients in the AA group had a higher mean systolic blood pressure (147.6 mm Hg) compared to those in the 4F-PCC group (139.9 mm Hg), which may have influenced the efficacy outcomes favoring AA. Similarly, Oh et al. [35] reported that despite higher baseline systolic pressures, AA demonstrated effective hemostatic control, which was crucial for patient survival and reduced mortality rates.

The ANNEXA-1 trial, which constitutes a substantial portion of the patient population in our meta-analysis, had specific inclusion criteria that could have influenced the outcomes observed. The trial included patients with relatively high baseline systolic blood pressure (mean 161.2 ± 27.0 mm Hg in the andexanet group) and higher initial Glasgow Coma Scale (GCS) scores, with a median GCS score of 15 in both treatment groups. Additionally, the trial excluded patients with a GCS score < 7 and those with a National Institutes of Health Stroke Scale score > 35, thereby selecting a cohort with potentially less severe neurological deficits at baseline. Furthermore, the strict time criteria, such as the requirement for the time from the onset of bleeding symptoms to the baseline imaging scan to be within 6 h, may have led to earlier intervention and better outcomes compared to other studies with more time windows. These criteria and the relatively favorable baseline characteristics could have contributed to the more pronounced efficacy observed in the ANNEXA-1 trial, thereby impacting the overall outcomes of our meta-analysis [18].

Hemostatic efficacy is an important parameter in the evaluation of reversal agents, Costa et al. [27] reported that AA was associated with better hemostatic effectiveness (85.8%) compared to 4F-PCC (68.1%), and these findings also align with those by Lipski et al. [33], who reported excellent efficacy in the favor of AA (66.7% for 4F-PCC vs. 75% for AA). The ANNEXA-4 trial [11] also strengthens the evidence base, as it shows AA’s ability to significantly reduce anti-FXaI activity and achieve high rates of excellent or good hemostatic efficacy. The efficacy of AA in most studies could be attributed to its targeted mechanism as a FXaI decoy receptor. However, it is important to note that the results in all our included studies were not significant, except for those in the ANNEXA-1 trial [18] and the study by Costa et al. [27], which may have deviated the overall effectiveness toward AA. To ensure the robustness of our results, we conducted a sensitivity analysis in multiple scenarios, excluding one study in each scenario (eAppendix 5).

Regarding the safety profile, especially thromboembolic complications, which represent another level of complexity in using either agent, most studies showed higher levels of thrombotic events with AA. However, in some studies, such as the study by Vestal et al. [17], higher events were detected in the 4F-PCC group (31.4%) compared to the AA group (14.3%). Although thromboembolic events were higher following the administration of AA in most of our included studies, all results showed no significant differences between both groups, except for the ANNEXA-1 trial [18], in which it showed a marginally significant difference (*P* = 0.048). The overall *P* value in our study was similar to that of the ANNEXA-1 trial (*P* = 0.046). In terms of mortality rates, most studies reported lower deaths in the AA group; Dobesh et al. [28] reported a significant reduction in odds of in-hospital mortality with AA compared to 4F-PCC, which suggests more survival benefits of AA in the setting of FXaI-related major bleeds. These findings are also supported by the findings of Sutton et al. [39], who observed a significantly lower in-hospital mortality rate in patients treated with AA compared to those receiving 4F-PCC.

The ANNEXA-1 trial [18] was the first randomized controlled trial to compare AA with usual care (including 4F-PCC in 87% of patients) in 530 patients receiving oral FXaI with ICH (mostly intracerebral). The primary outcome measured was hemostatic efficacy, defined as an expansion of hematoma volume by 35% or less at 12 h, an increase of less than 7 points on the National Institutes of Health Stroke Scale, and no receipt of rescue therapy. The results showed that hemostatic efficacy was achieved in 67.0% of patients in the AA group compared to 53.1% in the usual care group (*P* = 0.003). However, 30-day mortality rates were balanced between the treatment groups (*P* = 0.51). Thromboembolic events were more frequent in the AA group (10.3% versus 5.6% in the usual care group) (*P* = 0.048), with ischemic stroke occurring in 6.5% versus 1.5% of patients, respectively. When viewed together, the strong internal validity of the ANNEXA-1 trial for its primary end point and our pooled results suggests that AA is superior to usual care, including prothrombin complex concentrate, for improving hemostasis in patients with FXaI-associated ICH. Although the ANNEXA-1 trial was not designed to assess mRS scores and mortality rates, our results of 30-day mortality rates and mRS scores ≤ 3 showed no significant difference between the two groups, which was consistent with the ANNEXA-1 trial. However, our analysis favored AA in lowering the overall mortality rate (*P* = 0.02) and the in-hospital mortality rate, which was marginally significant (*P* = 0.047). Further randomized controlled trials powered to assess in-hospital and 30-day mortality rates and change in mRS scores from baseline as a primary end point are required to address this gap. Finally, the ANNEXA-1 trial found a marginally significant increase in thromboembolic events in the AA group (*P* = 0.048), which was consistent with our pooled overall effect (*P* = 0.046). The mechanism of thrombotic events in AA is still uncertain.

Several international and national medical associations have established recommendations on the optimal management of ICH associated with FXaI. The current guidelines from the American College of Cardiology [40], European Society of Cardiology [41], European Stroke Organization [42], American College of Emergency Physicians [43], and the American Society of Hematology [44] all advocate for the use of AA in addressing severe life-threatening bleeding associated with apixaban or rivaroxaban use. Some of them [41, 42] suggest the use of 4F-PCC as an alternative when AA is not available or for non-life-threatening bleeds, although this recommendation is not universally supported.

Previous studies showed conflicting results of cost-effectiveness models to assess whether the better results of AA in improving hemostatic efficacy are enough to make up for their higher cost and low availability. The study by Keinath et al. [45] underscores the financial aspect of choosing between AA and 4F-PCC for reversing FXaI-associated bleeds. Their findings indicate no significant difference in clinical outcomes between AA and 4F-PCC. However, AA’s cost was notably higher, approximately four times the cost per deterioration-free discharge compared to 4F-PCC. The cost per deterioration-free discharge was $20,773.62 in the AA group versus $5,230.32 in the 4F-PCC group. The study by Pham et al. [15] found a statistically significant difference in the total cost of treatment between the two agents: $23,602 for treatment with AA and $6,692 for treatment with 4F-PCC. This economic disparity is a pivotal factor in our discussion, highlighting the necessity of weighing both clinical efficacy and cost-effectiveness in treatment decisions. Another challenge associated with the use of AA is the long time from door to reversal administration compared to 4F-PCC. This may be attributed to the increased medication compounding complexity outside the emergency department setting. However, because of its short duration of action compared to 4F-PCC, periprocedural use must be optimized carefully to promote intraoperative and postoperative hemostasis [29].

Our study has several limitations. One limitation is the geographical uniformity of the included studies, as all these studies were conducted in the USA, which may limit the generalizability of our findings. Moreover, the baseline blood pressure and door-to-reversal time were not reported in most studies, which could limit the robustness of the conclusions drawn. Also, although the majority of the included studies employed the criteria established by the International Society of Thrombosis and Hemostasis to evaluate the efficacy of managing significant bleeding events [46], few studies used predetermined adjudication criteria or failed to describe the specific criteria used to determine the stability of ICH on repetitive imaging (eAppendix 4). In addition, it is important to note that all the comparisons are restricted to data from cohort studies, except for the ANNEXA-1 trial [18]. Additionally, the ANNEXA-1 trial, which contributed a significant proportion of the patients included in our analysis, was not designed with sufficient power to detect differences in mortality and mRS outcomes. This limitation may affect the generalizability of our results. Finally, the inclusion of only a limited number of studies reporting mRS and GOS outcomes restricts our ability to fully assess the short-term and long-term clinical impact of the interventions studied. Nonetheless, to the best of our knowledge, our meta-analysis is the first to conclude significant results favoring AA over 4F-PCC in the context of reversing anticoagulation in FXaI-associated ICH.

## Conclusions

Our systematic review and meta-analysis of 16 studies with 2,977 patients demonstrated that AA is superior to 4F-PCC in reversing anticoagulation in patients with ICH on FXaI. Lower mortality rates were significantly found following AA administration, with more thromboembolic events in the AA group. Our findings suggest the potential use of AA as a preferred reversal agent in acute ICH. However, its use as a reversal agent may be limited because of its cost and low availability in low-resource hospitals, which potentially influences treatment decisions for ICH associated with FXaI.

## Supplementary Information

Below is the link to the electronic supplementary material.

Supplementary file 1 (DOCX 174 kb)

## Data Availability

All relevant data are available within the article and supplementary files.
