# Supplementary material for: Efficacy and Safety of Andexanet Alfa Versus Four Factor Prothrombin Complex Concentrate for Emergent Reversal of Factor Xa Inhibitor Associated Intracranial Hemorrhage: A Systematic Review and Meta-Analysis
Source: Neurocrit Care. 2024 Oct 8;42(2):701–14. doi: 10.1007/s12028-024-02130-y (PMC11950062; doi:10.1007/s12028-024-02130-y)

**Supplementary Online Content**

**Efficacy and Safety of Andexanet Alpha Versus Four Factor Prothrombin Complex Concentrate for Emergent Reversal of Factor Xa Inhibitor Associated Intracranial Hemorrhage: A Systematic Review and Meta-Analysis**

**eAppendix 1.** Search Strategy

**eAppendix 2.** Summary of the Included Studies

**eAppendix 3.** Indication for Anticoagulation

**eAppendix 4.** Definitions of Hemostasis (Successful Anticoagulation Reversal) in the Included Studies

**eAppendix 5.** Sensitivity analysis for Primary and Secondary Outcomes

**eAppendix 6.** Funnel Plots Assessing Publication Bias for Primary and Secondary Outcomes

**eAppendix 1. Search Strategy**

(("Prothrombin complex concentrates"[Title/Abstract]) OR (PCC[Title/Abstract]) OR (Cofact[Title/Abstract]) OR ( Beriplex[Title/Abstract] ) OR ( Confidex[Title/Abstract] ) OR ( Octaplex[Title/Abstract] ) OR ( Kcentra[Title/Abstract] ) OR ( PPSB[Title/Abstract] ) OR ( "PPSB SD"[Title/Abstract] ) OR ( "Prothrombinex-VF"[Title/Abstract] ) OR ( Prothrombinex[Title/Abstract] ) OR ( "prothrombin complex"[Title/Abstract] ) OR ( "blood coagulation factors"[Title/Abstract] ) OR ( "blood Coagulation Disorders"[Title/Abstract] ) OR ( "4-factor"[Title/Abstract] ) OR ( "four-factor"[Title/Abstract] ) OR ( "4F-PCC"[Title/Abstract] ) OR ( "4-PCC"[Title/Abstract] ) OR ( PCC4[Title/Abstract] )) AND ("Factor Xa Inhibitor*"[Title/Abstract]) OR ("Rivaroxaban"[Title/Abstract]) OR ("Rivaroxaban"[Mesh]) OR ("Xarelto" [Title/Abstract]) OR ("apixaban" [Title/Abstract]) OR ("Eliquis"[ Title/Abstract]) OR ("edoxaban" [Title/Abstract]) ("Savaysa"[ Title/Abstract]) OR ("betrixaban" [Title/Abstract]) OR ("Direct oral anticoagul*"[ Title/Abstract]) OR ("DOAC*"[ Title/Abstract]) OR ("Direct Acting Oral Anticoagulant*"[ Title/Abstract]) OR ("Novel Oral anticoagul*"[ Title/Abstract]) OR ("NOAC*"[ Title/Abstract]) OR ("non-vitamin K oral anticoagul*"[ Title/Abstract]) AND (("severe injur*"[Title/Abstract]) OR (severe trauma[Title/Abstract]) OR (multiple injur*[Title/Abstract]) OR (multiple trauma[ Title/Abstract]) OR (major trauma[Title/Abstract]) (major injur*[ Title/Abstract]) OR (multiple trauma[Mesh]) OR (critical bleed*[ Title/Abstract]) OR (post trauma haemorrhage[ Title/Abstract]) OR (post trauma hemorrhage [Title/Abstract]) OR (intracranial hemorrhage[ Title/Abstract]) OR (hemorrhagic injury[ Title/Abstract]) OR (''Traumatic brain injury''[ Title/Abstract]) OR (Bleeding[ Title/Abstract]) OR (Hemorrhage[Title/Abstract])) AND ((andexanet alfa [Title/Abstract]) OR (PRT064445[Title/Abstract]))

*Translations:*

1. (("Prothrombin complex concentrates"[Title/Abstract]) OR (PCC[Title/Abstract]) OR (Cofact[Title/Abstract]) OR ( Beriplex[Title/Abstract] ) OR ( Confidex[Title/Abstract] ) OR ( Octaplex[Title/Abstract] ) OR ( Kcentra[Title/Abstract] ) OR ( PPSB[Title/Abstract] ) OR ( "PPSB SD"[Title/Abstract] ) OR ( "Prothrombinex-VF"[Title/Abstract] ) OR ( Prothrombinex[Title/Abstract] ) OR ( "prothrombin complex"[Title/Abstract] ) OR ( "blood coagulation factors"[Title/Abstract] ) OR ( "blood Coagulation Disorders"[Title/Abstract] ) OR ( "4-factor"[Title/Abstract] ) OR ( "four-factor"[Title/Abstract] ) OR ( "4F-PCC"[Title/Abstract] ) OR ( "4-PCC"[Title/Abstract] ) OR ( PCC4[Title/Abstract] ))
2. ("Factor Xa Inhibitor*"[Title/Abstract]) OR ("Rivaroxaban"[Title/Abstract]) OR ("Rivaroxaban"[Mesh]) OR ("Xarelto" [Title/Abstract]) OR ("apixaban" [Title/Abstract]) OR ("Eliquis"[ Title/Abstract]) OR ("edoxaban" [Title/Abstract]) ("Savaysa"[ Title/Abstract]) OR ("betrixaban" [Title/Abstract]) OR ("Direct oral anticoagul*"[ Title/Abstract]) OR ("DOAC*"[ Title/Abstract]) OR ("Direct Acting Oral Anticoagulant*"[ Title/Abstract]) OR ("Novel Oral anticoagul*"[ Title/Abstract]) OR ("NOAC*"[ Title/Abstract]) OR ("non-vitamin K oral anticoagul*"[ Title/Abstract])
3. (("severe injur*"[Title/Abstract]) OR (severe trauma[Title/Abstract]) OR (multiple injur*[Title/Abstract]) OR (multiple trauma[ Title/Abstract]) OR (major trauma[Title/Abstract]) (major injur*[ Title/Abstract]) OR (multiple trauma[Mesh]) OR (critical bleed*[ Title/Abstract]) OR (post trauma haemorrhage[ Title/Abstract]) OR (post trauma hemorrhage [Title/Abstract]) OR (intracranial hemorrhage[ Title/Abstract]) OR (hemorrhagic injury[ Title/Abstract]) OR (''Traumatic brain injury''[ Title/Abstract]) OR (Bleeding[ Title/Abstract]) OR (Hemorrhage[Title/Abstract]))
4. ((andexanet alfa [Title/Abstract]) OR (PRT064445[Title/Abstract]))

**eAppendix 2.** Summary of the Included Studies

| **Study ID** | **Study design** | **Country** | **Inclusion Criteria** | **AA Dose** | **4F-PCC Dose** | **Factor XaI** | **Main findings** | | **Risk of bias** |
| --- | --- | --- | --- | --- | --- | --- | --- | --- | --- |
| Ammar 2021 | Retrospective study | USA | 1-Adult patients, 2-life-threatening intracranial bleeds taking FXaI (apixaban or rivaroxaban), 3-Patients treated with either at least one dose of AA or 4F-PCC | According to the product labeling for life-threatening bleeding associated with factor Xa inhibitors | 25 units/kg up to 2,500 units per dose. | Apixaban or Rivaroxaban | No difference between AA and 4F-PCC regarding outcomes measured | | High |
| ANNEXA-1 2024 | RCT |  | 1- Age ≥ 18 years old 2- An acute intracerebral bleeding episode defined as an estimated blood volume ≥ 0.5 to ≤ 60 mL acutely observed radiographically within the cerebrum. 3- Performance of a head CT or MRI scan demonstrating the intracerebral bleeding within 2 hours prior to randomization. 4- Treatment with an oral FXa inhibitor (apixaban [last dose 2.5 mg or greater], rivaroxaban [last dose 10 mg or greater], or edoxaban [last dose 30 mg or greater]. 5- Time from bleeding symptom onset < 6 hours prior to the baseline imaging scan 6- NIHSS score ≤ 35 at the time of consent. | Low: Initial 400 mg at a target rate of 30 mg/min for ~15 minutes followed by 480 mg at a target rate of 4 mg/min for 120 minutes.  High: Initial 800 mg at a target rate of 30 mg/min for up to ~30 minutes followed by 960 mg at a target rate of 8 mg/min for 120 minutes. | NR | Apixaban or Rivaroxaban or Edoxaban | AA resulted in better control of hematoma expansion than usual care but was associated with thrombotic events, including ischemic stroke. | | Low |
| Barra 2020 | Retrospective study | USA | 1- 18 years or older patients 2-patients were on rivaroxaban or apixaban therapy at baseline, 3-patients received AA or 4F-PCC for reversal of ICH. | Low-dose: 400 mg IV bolus over 15 minutes followed by 480 mg infused over 2 hours, High-dose: 800 mg IV bolus over 30 minutes followed by 960 mg infused over 2 hours | 25-50 units/kg, max dose 5000 units | Apixaban or Rivaroxaban | Patients who received AA showed higher rates of occurrence of good or excellent hemostasis and GOS > 3 on hospital discharge and increased incidence of thrombosis compared to 4F-PCC. | | High |
| Coleman 2020 | Retrospective study | USA | 1-Adult patients. 2-patients specifically received a FXaI prior to hospital admission. | NR | NR | Apixaban, Rivaroxaban, Betrixaban, or Edoxaban | AA showed the lowest mortality rate across all types of bleeding and resulted in a shorter stay in the intensive care unit when compared 4F-PCC | | High |
| Costa 2022 | Propensity score-overlap  weighted analysis | USA | 1- 18 years and older, 2- Admitted for a radiographically confirmed acute ICH, 3- patients have taken apixaban or rivaroxaban within 24 h of the bleed. | **Low-dose:** 400 mg bolus+440 mg infusion, **High-dose:** 800 mg bolus+860 mg infusion | 25 units/kg infusion, 50 units/kg infusion | Apixaban or Rivaroxaban | AA was associated with better hemostatic effectiveness and improved survival compared to 4F-PCC | | Low |
| Dobesh 2023 | Retrospective study | USA | 1- 18 years or older, 2-patients were taking either rivaroxaban or apixaban at the time of the bleeding event, 3- patients treated with either AA or 4F-PCC during the index hospitalization, | **Low dose:** 400 mg bolus (delivered at a target rate of 30 mg/min, followed by a 4 mg/min infusion over 120 min), **High dose:** 800 mg bolus (delivered at a target rate of 30 mg/min, followed by an 8 mg/min infusion over 120 min). | mean of 2510 total units were administered per patient | Apixaban or Rivaroxaban | AA was associated with lower in-hospital mortality than 4F-PCC | | Low |
| Koo 2024 | Retrospective study | USA | 1- Adult subjects (18 years or older) 2- Admitted for a DOAC-associated major bleeding 3- Received 4F-PCC or AA | FDA-approved dose (high or low) | Fixed dosing of 2000 units or weight-based dosing (25-50 units/kg) IV with a maximum of 5000 units | Apixaban or Rivaroxaban | There were no significant differences between 4F-PCC and AA according to outcomes measured | | High |
| Irizarry-Gatell, 2024 | Retrospective study | USA | 1- Greater than 18 years of age 2- Had life-threatening ICH that required ICU level care 3- Received at least one dose of apixaban or rivaroxaban prior to presentation 4- Received a dose of 4F-PCC or Andexanet alfa | According to the product labeling for life-threatening bleeding associated with factor Xa inhibitors | Doses ranged from 1048 units to 9448 units with a median dose of 49 units/kg [IQR 45–51] | Apixaban or Rivaroxaban | No clinical differences were observed with respect to selection of reversal agent | | High |
| Lipski 2023 | Retrospective study | USA | 1-18 years old or older 2-patients received 4F-PCC or AA 3-patients were on apixaban or rivaroxaban with associated ICH | According to the product labeling for life-threatening bleeding associated with factor Xa inhibitors | 50 IU/kg with a maximum dose of 5000 IU | Apixaban or Rivaroxaban | No significant difference between 4F-PCC or AA in hemostatic efficacy | | High |
| Miliogloua 2022 | Retrospective study | USA | 1-18 years or older patients who were receiving FXaI. 2- patients presented with any ICH 3- Patients administered either AA or 4F-PCC. | **Low dose:** 400 mg IV bolus administered at 30 mg/min, followed 2 min later by 4 mg/min IV infusion for up to 120 min, **High dose:** 800 mg IV bolus administered at 30 mg/min, followed 2 min later by 8 mg/minute IV infusion for up to 120 min | 25–50 units/kg of actual body weight | Apixaban or Rivaroxaban | Mortality at discharge appears to be similar between the AA and 4F-PCC group. | | High |
| Oh 2023 | Retrospective study | USA | 1-Patients 18 years of age or older 2- received AA or 4F-PCC for the reversal of apixaban or rivaroxaban within 18 h in the setting of an intracranial hemorrhage ICH. | **low dose:** 400 mg intravenous bolus over 15 min followed by 480 mg intravenous over 2 h, **High dose:** 800 mg intravenous bolus over 30 min followed by 960 mg intravenous over 2 h | 50 units/kg intravenous once | Apixaban or Rivaroxaban | Administration of AA and 4F-PCC to reverse factor Xa inhibitors associated with intracranial hemorrhage reveals similar real-world clinical and safety outcomes as observed in clinical trials. | | High |
| Parsels 2022 | Retrospective study | USA | patients took AA or 4F-PCC for reversal of apixaban, or rivaroxaban associated new ICH on CT or MRI. | Low-dose or high-dose AA were recommended based on manufacturer criteria beginning in 2019 | 25–50 units/kg per actual body weight | Apixaban or Rivaroxaban | No significant differences in ICH hemostasis or new thrombotic events between AA and 4F-PCC | | High |
| Pham 2022 | Retrospective study | USA | 1-Patients aged 18 years or older with 2-Documented neuroimaging of ICH. 3-Documented home medication of apixaban or rivaroxaban. 4-Administration of either AA or 4F-PCC. | AA was dosed according to the product labeling for life-threatening bleeding associated with factor Xa inhibitors. | 50 units/kg (max 5000 units) for one dose. | Apixaban or Rivaroxaban | There was no significant difference observed between (AA) and (4F-PCC) in achieving excellent hemostasis or secondary outcomes. | | High |
| Stevens 2021 | Retrospective study | USA | ≥18 years old patients who had received 4F-PCC or AA for the reversal of Apixaban or Rivaroxaban-related bleeding | **Low dose:** 400 mg IV bolus followed by 480 mg IV infusion over 2h, **High dose:** 800 mg intravenous (IV) bolus followed by 960 mg IV infusion over  2 h | 27.9 ± 11.7 units/kg | Apixaban or Rivaroxaban | AA and 4F-PCC achieved similar effective hemostasis in most patients. | | High |
| Troyer 2023 | Retrospective study | USA | 1- Patients 18 years of age or older 2- diagnosed with ICH and received either 4FPCC or AA for reversal of apixaban or rivaroxaban. | **Low dose:** 400 mg intravenous bolus over 15 min followed by 480 mg intravenous over 2 h, **High dose:** 800 mg intravenous bolus over 30 min followed by 960 mg intravenous over 2 h | Standardized weight-based dosing of 50 units per kilogram up to 5000 units | Apixaban or Rivaroxaban | No significant difference in hemostatic efficacy was found between AA and 4FPCC. | | High |
| Vestal 2021 | Retrospective study | USA | 1- Patients ≥18 years of age diagnosed with ICH. 2- patients were anticoagulated with apixaban or rivaroxaban prior to presentation. 3- Received either AA or 4F-PCC | **Low dose:** 400 mg IV bolus+480 mg infusion, **High dose:** 800 mg IV bolus+960 mg infusion | 50 units/kg (Max: 5000 units) | Apixaban or Rivaroxaban | Higher rate of thrombotic events and mortality was reported in 4F-PCC group compared to AA. | | High |
| *AA* Andexanet Alpha. *4F-PCC* 4 factor prothrombin complex concentrate. *ICH* intracranial hemorrhage. *NR* not reported. | | | | | | | |  |  |

**eAppendix 3. Indication for Anticoagulation**

| **Study ID** | **Indication for Anticoagulation** | | | | | |
| --- | --- | --- | --- | --- | --- | --- |
|  | **Atrial Fibrillation (%)** | | **Venous Thromboembolism (%)** | | **Others (%)** | |
|  | **AA** | **4F-PCC** | **AA** | **4F-PCC** | **AA** | **4F-PCC** |
| **Ammar 2021** | 75% | 81% | 21% | 19% | 4% | 0% |
| **ANNEXA-1 2024** | 87.5% | 84.6% | 7% | 12% | 5% | 3% |
| **Barra 2020** | 94.40% | 72.70% | 11.10% | 27.30% | 0% | 0% |
| **Coleman 2020** | NA | NA | NA | NA | NA | NA |
| **Costa 2022** | 87.90% | 82.10% | NA | NA | NA | NA |
| **Dobesh 2023** | NA | NA | NA | NA | NA | NA |
| **Irizarry-Gatell 2024** | 78% | 82% | 9% | 18% | 13% | 0% |
| **Koo 2024** | 78.60% | 87.90% | 22.60% | 7% | 3.60% | 5% |
| **Lipski 2023** | 65.20% | 68.10% | 26.10% | 25.50% | 8.70% | 6.40% |
| **Miliogloua 2022** | 87% | 90.90% | 13% | 9.10% | 0% | 0% |
| **Oh 2023** | NA | NA | NA | NA | NA | NA |
| **Parsels 2022** | 77% | 76.90% | 23% | 23.10% | 0% | 0% |
| **Pham 2022** | 83% | 77.40% | 13% | 16.10% | 4% | 6.50% |
| **Stevens 2021** | 34% | 43.80% | 16% | 6.30% | 50% | 50% |
| **Troyer 2023** | 67.70% | 60% | 25.80% | 26.70% | 6.50% | 13.30% |
| **Vestal 2021** | 67% | 71.40% | 33% | 25.70% | 0% | 2.90% |

**eAppendix 4.** **Hemostasis Definition in the Included Studies**

| **Study ID** | **Hemostasis definition on brain imaging** |
| --- | --- |
| **Ammar, 2021** | Stable head computed tomography (CT) scan at 6 and 24 h post-administration of AA or 4F-PCC, defined for IPH as no significant increase in volume (less than 6 mL or 33% of baseline volume).  Stability was defined as a similar amount of blood from one scan to the next.  For intraparenchymal hemorrhages, the volume of the hematoma was calculated using the ABC/2 volume estimation method. In IPH, a similar amount of blood was defined as a volume growth of less than 6 mL or 33% from baseline CT and adjudicated by the three experienced independent providers. |
| **ANNEXA-1, 2024** | Hemostatic efficacy was defined in the trial as an expansion of the hematoma volume of 35% or less at 12 hours, an increase of less than 7 points on the National Institutes of Health Stroke Scale (NIHSS; scores range from 0 to 42, with higher scores indicating worse neurologic deficit), and no receipt of rescue therapy) and the absence of rescue therapies such as Andexanet, prothrombin complex concentrate, or surgery to decompress the hematoma within 3 to 12 hours after randomization. |
| **Barra, 2020** | Hemostasis was defined based on the ANNEXA-4 |
| **Coleman, 2020** | No data available to evaluate ICH hemostasis |
| **Costa, 2022** | Hemostasis was defined based on the ANNEXA-4 |
| **Dobesh, 2023** | Study outcomes were not concerned with hemostatic efficacy |
| **Irizarry-Gatell 2024** | Radiographic stability of bleed, defined as stable from reversal agent to follow up imaging. |
| **Koo 2024** | Hemostasis was defined based on the ANNEXA-4 |
| **Lipski 2023** | Hemostasis was defined based on the ANNEXA-4 |
| **Miliogloua, 2022** | Brain imaging on presentation and repeat imaging within the first 24-or 48-h were assessed. Hemostasis effectiveness was evaluated by two physicians who reviewed separately all CT and/or MRI results before and after intervention (PCC or andexanet). Differences between brain hemorrhage volume estimates between the two physicians were resolved by discussion. No further information provided. |
| **Oh 2023** | Hemostasis was defined based on the ANNEXA-4  . |
| **Parsels, 2022** | Hemostasis was defined based on the ANNEXA-4 |
| **Pham 2022** | Hemostasis was defined based on the ANNEXA-4 |
| **Stevens, 2021** | Hemostasis was defined based on the ANNEXA-4 |
| **Troyer, 2022** | Hemostasis was defined based on the ANNEXA-4  . |
| **Vestal,2021** | Hemostatic efficacy was determined by radiologists’ interpretations of the diagnostic and first subsequent head scans. If radiologist’s report stated an increase in hematoma volume, hemostatic efficacy was labeled as “No”. If no progression was noted by the interpreting radiologist, hemostatic efficacy was labeled as “Yes”. In terms of imaging, it was the standard institutional clinical practice to obtain a repeat scan six hours after the initial scan to assess progression of the hematoma. |
| *ANNEXA-4 defined excellent or good hemostatic efficacy as hematoma volume expansion for ICH of ≤35%. | |

**eAppendix 5.** **Sensitivity analysis for Primary and Secondary Outcomes**

A) Anticoagulation Reversal


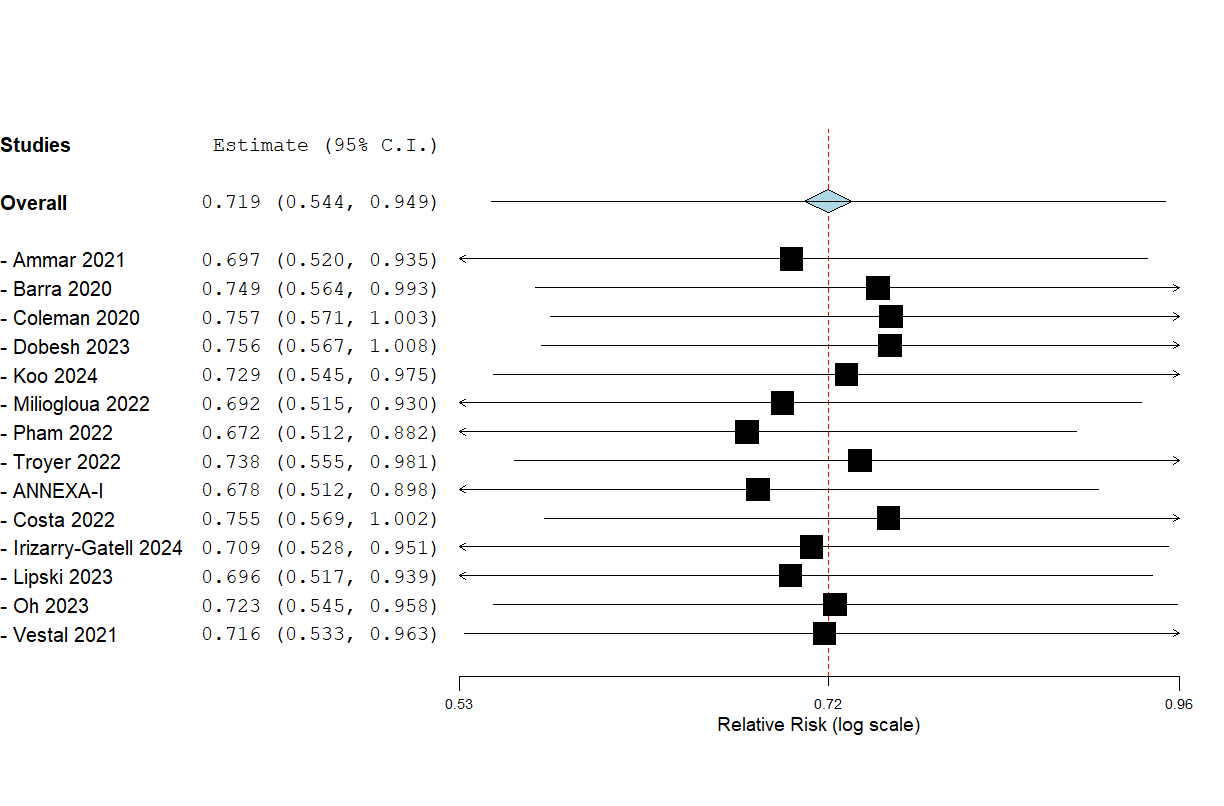


B) Mortality


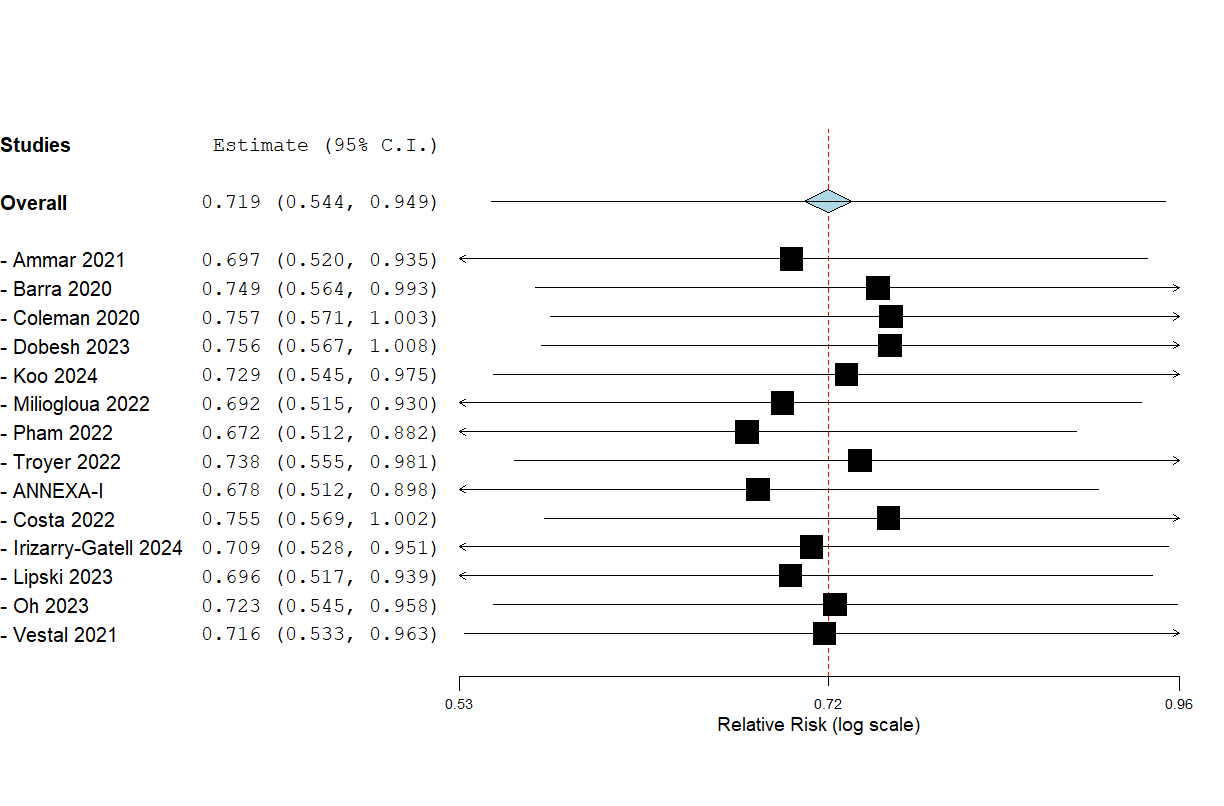


C) Thromboembolic events


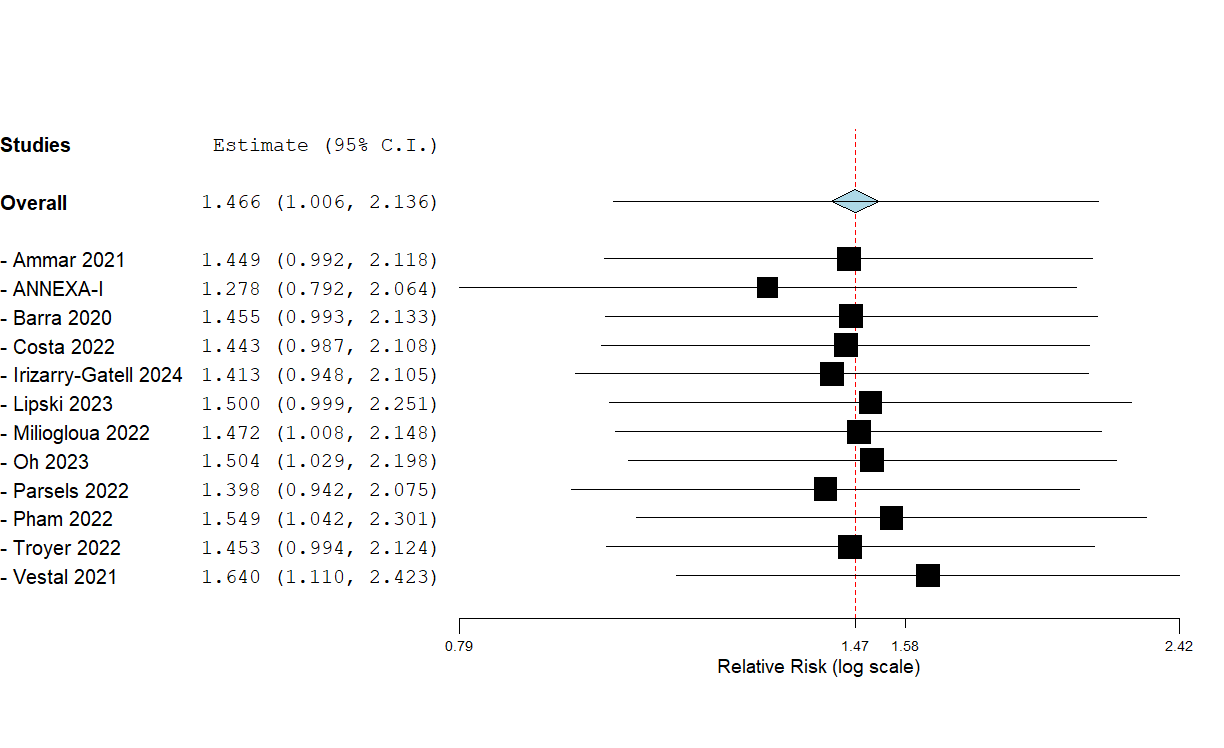


D) Length of Hospital Stay


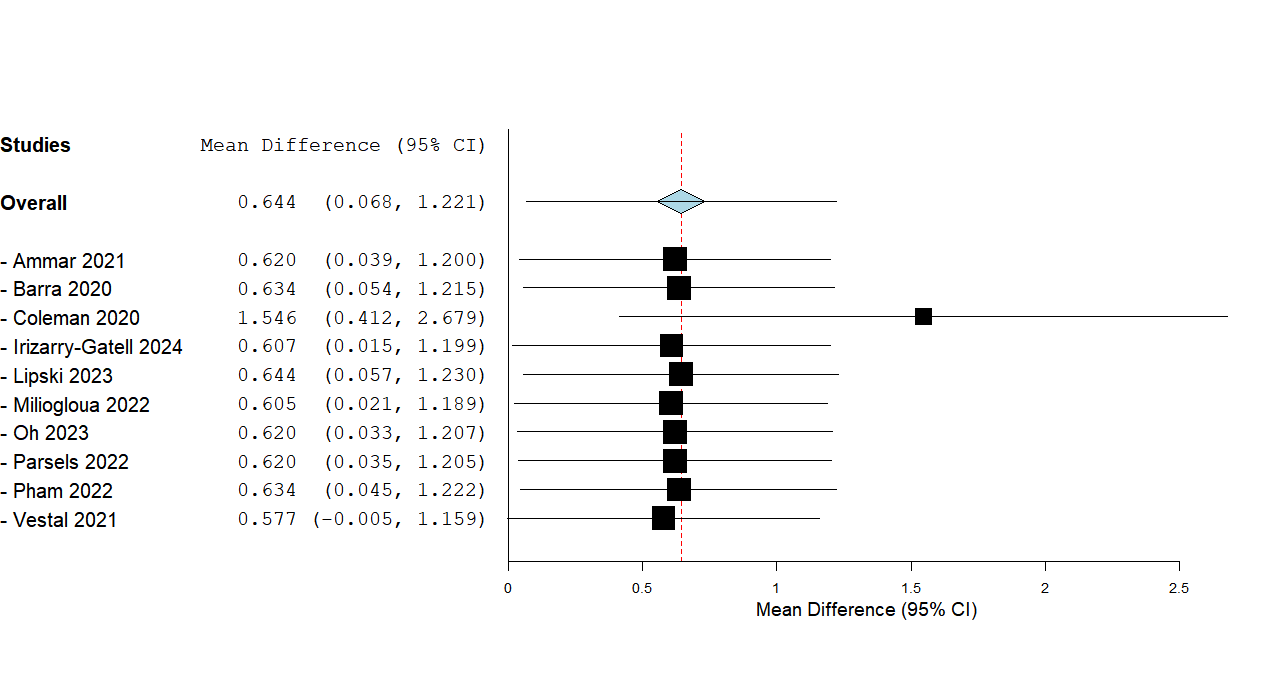


E) Length of ICU stay


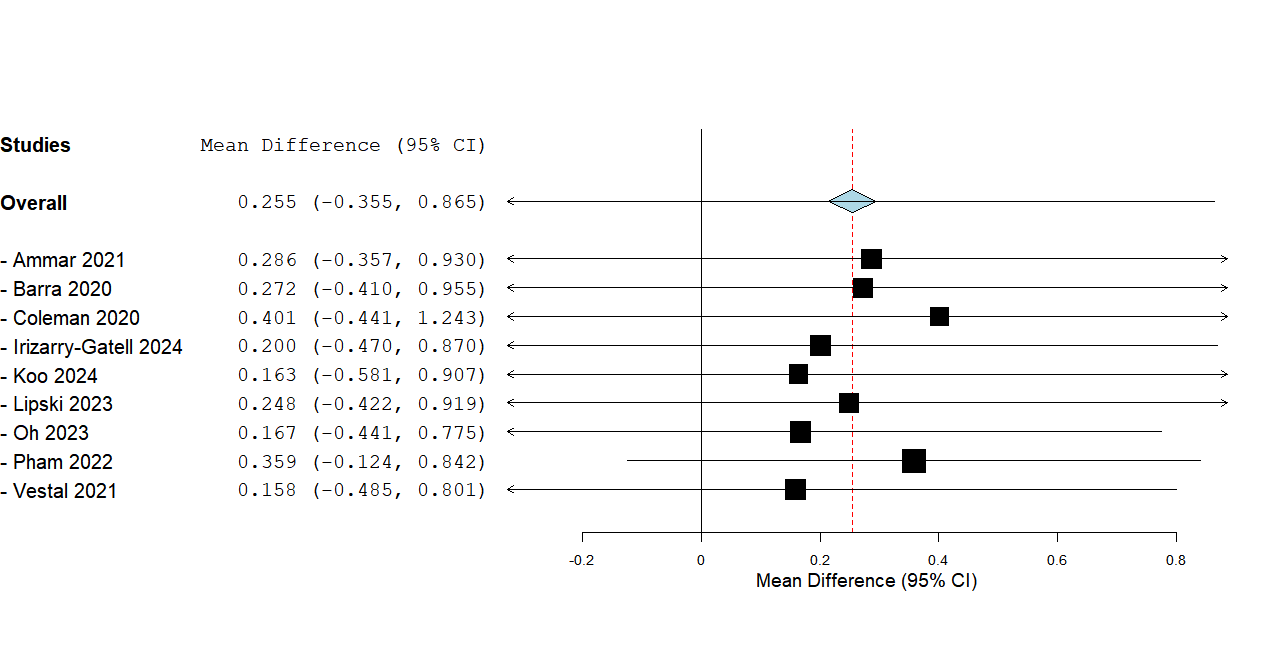


F) Hematoma Volume Expansion


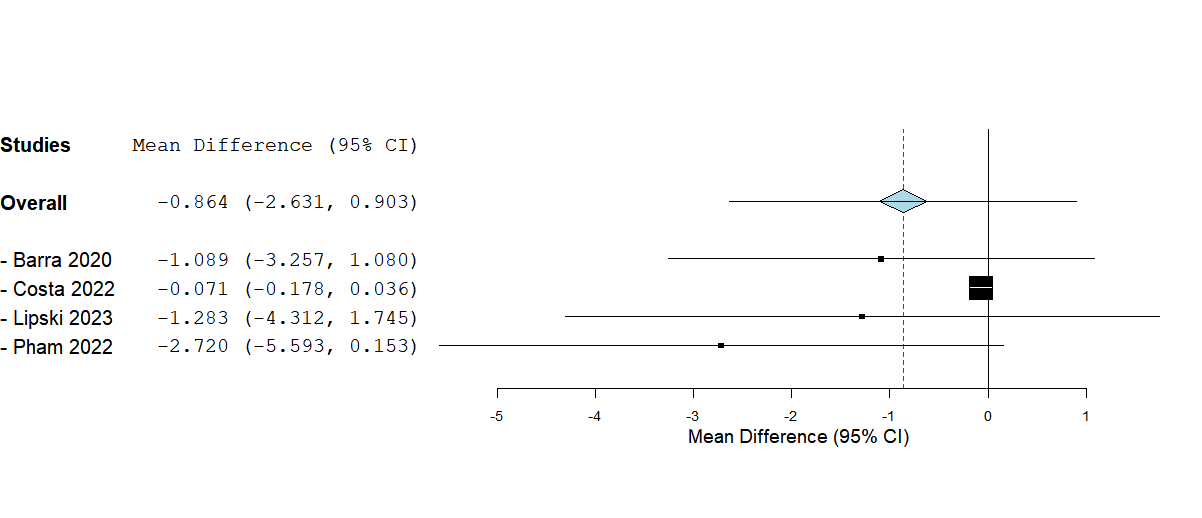


G) Good Clinical Outcome (mRS ≤ 3 or GOS > 3)


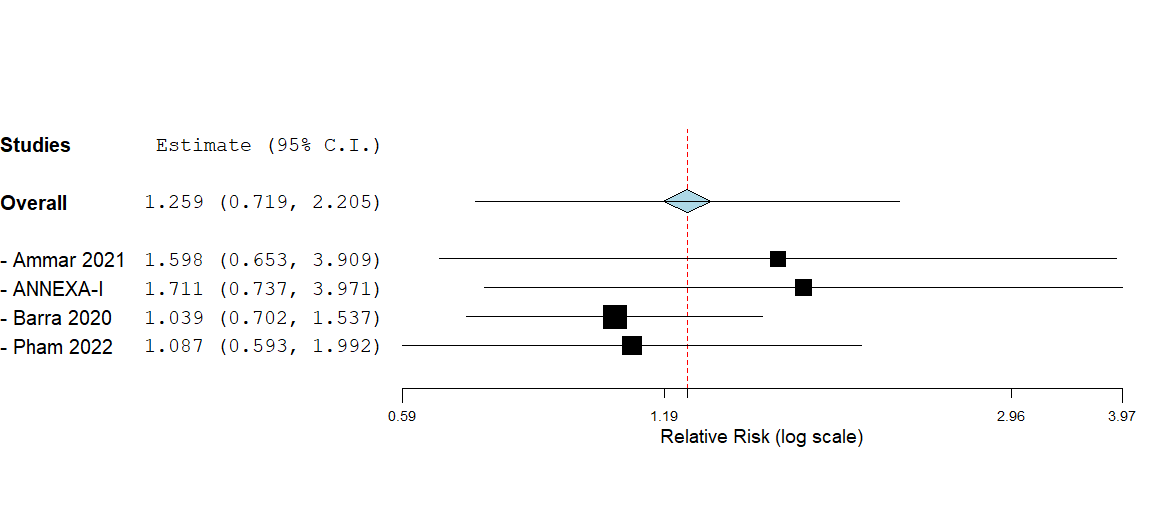


**eAppendix 6.** **Funnel Plots Assessing Publication Bias for Primary and Secondary Outcomes**

A) Anticoagulation Reversal


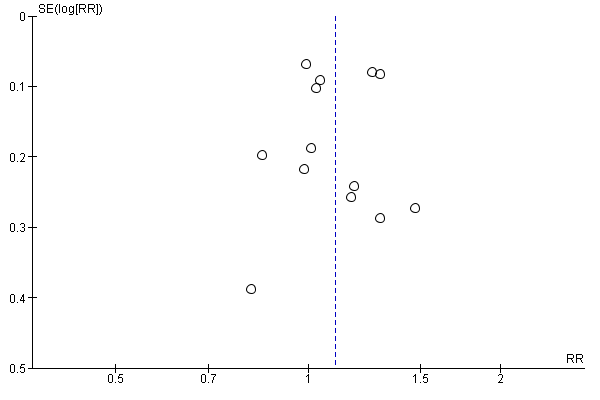


B) Mortality


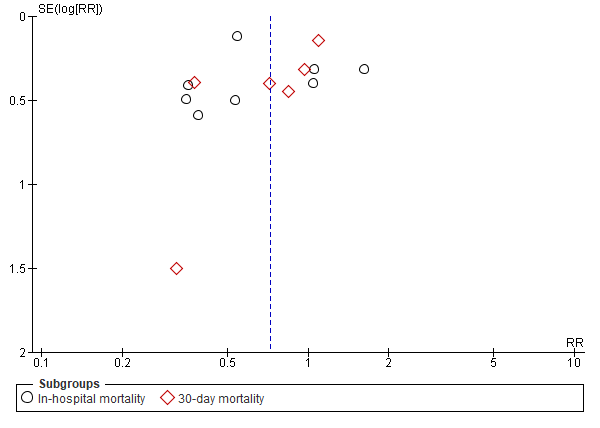


C) Thromboembolic Events


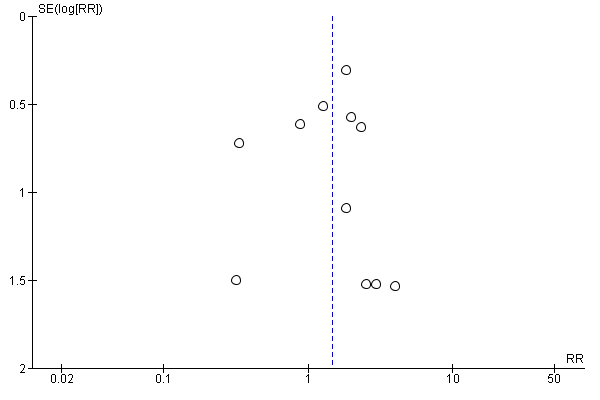


D) Length of Hospital Stay


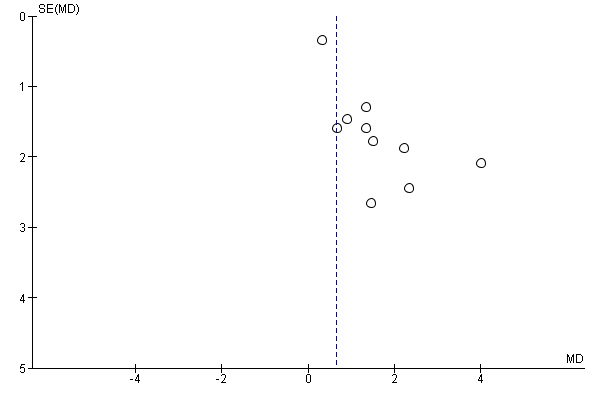


E) Length of ICU Stay


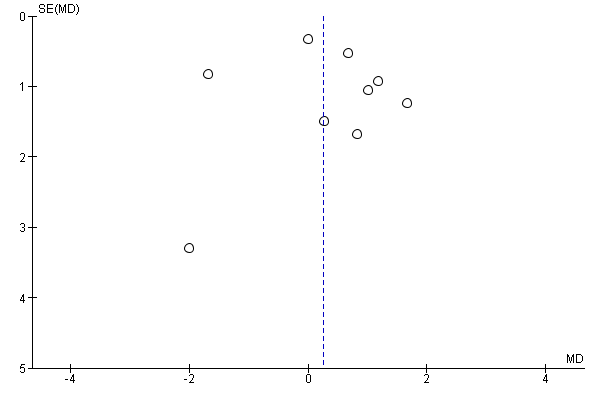

Supplement: Supplementary file 1 — (DOCX 174 kb) [file 12028_2024_2130_MOESM1_ESM.docx]
